# West Nile or Usutu Virus? A Three-Year Follow-Up of Humoral and Cellular Response in a Group of Asymptomatic Blood Donors

**DOI:** 10.3390/v12020157

**Published:** 2020-01-29

**Authors:** Elena Percivalle, Irene Cassaniti, Antonella Sarasini, Francesca Rovida, Kodjo Messan Guy Adzasehoun, Ilaria Colombini, Paola Isernia, Irene Cuppari, Fausto Baldanti

**Affiliations:** 1 Molecular Virology Unit, Microbiology and Virology Department, IRCCS Policlinico San Matteo, Via Taramelli 5, 27100 Pavia, Italy; e.percivalle@smatteo.pv.it (E.P.); i.cassaniti@smatteo.pv.it (I.C.); a.sarasini@smatteo.pv.it (A.S.); f.rovida@smatteo.pv.it (F.R.); guyadzase@yahoo.fr (K.M.G.A.); 2 SIMT, Centro Lavorazione e Validazione, Ospedale di Cremona, 26100 Cremona, Italy; i.colombini@asst-cremona.it; 3 SIMT, Centro Lavorazione e Validazione, IRCCS Policlinico San Matteo Pavia, 27100 Pavia, Italy; p.isernia@smatteo.pv.it; 4 SIMT, Centro Lavorazione e Validazione, Ospedale Maggiore Milano, 20162 Milano, Italy; irene.cuppari@ospedaleniguarda.it; 5 Department of Clinical, Surgical, Diagnostic and Pediatric Sciences, University of Pavia, 27100 Pavia, Italy

**Keywords:** blood donors, West Nile virus, Usutu virus, flavivirus, Lombardia region

## Abstract

West Nile virus (WNV) and Usutu virus (USUV) are two related arboviruses (genus *Flavivirus*, family *Flaviviridae*), with birds as a reservoir and mosquitoes as transmitting vectors. In recent years, WNV epidemiology changed in many European countries with increased frequency of outbreaks posing the issue of virus transmission risks by blood transfusion. USUV emerged for the first time in birds of the Tuscany region (Italy) in 1996 and in 2001 in Austria. While WNV is responsible for both mild and neuroinvasive diseases, USUV infection is usually asymptomatic and neuroinvasive symptoms are rare. Since WNV and USUV co-circulate, the surveillance of WNV allows also the detection of USUV. Due to the great similarity in amino-acid sequence of major surface proteins of the two viruses, a high cross-reactivity can lead to misinterpretation of serological results. Here, we report the results obtained from 54 asymptomatic blood donors during a three-year follow-up showing an unexpected high positivity (46.3%) for USUV. The major obstacle encountered in the differential diagnosis between these two viruses was the high cross-reactivity found in neutralizing antibodies (NT Abs) and, in some cases, a long follow-up was mandatory for a correct diagnosis. Moreover, two new ELISpot assays were developed for a more rapid and specific differential diagnosis, especially in those cases in which NT Abs were not determinant. Using a combination of Enzyme-linked immunospot (ELISpot), molecular, and serological tests, we could identify 25 true positive WNV and 25 true positive USUV blood donors. Our data highlight the importance of raising awareness for increasing USUV infections in endemic countries involved in blood transfusion and organ donation.

## 1. Introduction

West Nile virus (WNV) is an arbovirus (genus *Flavivirus*, family *Flaviviridae*), transmitted to humans by mosquito bite in a cycle in which different species of birds act as reservoir. Infection in humans is mainly asymptomatic (80%), while West Nile fever (WNF) is observed in 20% of cases and West Nile neuroinvasive disease (WNND) in less than 1% of cases [1]. 

In Italy (Tuscany region), the presence of WNV was reported in horses since 1998 [2]. Until 2007, sporadic cases and self-limited outbreaks were reported [3]. From 2008, the increased number of WNV infections in humans in Emilia Romagna and Veneto regions led to the introduction of an integrated surveillance system for vectors and birds in order to rapidly identify WNV circulation and reduce the blood donor screening only to those areas in which the virus was circulating [4,5]. These preventive measures were rapidly extended in many Italian regions [6]. 

Usutu virus (USUV) is a WNV-related flavivirus, which appeared for the first time in wild birds in the Tuscany region (Italy), in 1996 [7], and in 2001 in Austria [8]. Although USUV rarely causes human disease, in 2009, two severe cases were reported in immunocompromised patients [9,10]. Since WNV and USUV showed simultaneous circulation [11] and similar transmission cycles [12], WNV surveillance also allows the detection of USUV. However, the great similarity in the amino-acid sequence of major surface proteins of the two viruses is responsible for the high cross-reactivity that may lead to misinterpretation of serological results [13,14,15]. Moreover, conflicting results were reported on seroprevalence and duration of immunity for WNV [16,17], whereas less is known for USUV seroprevalence and immunity.

From a molecular point of view, to date, the automated WNV nucleic acid test (NAT) test also detects USUV-positive blood donors [18], but it is not able to distinguish between these two flaviviruses. This fact can generate erroneous statistics on the incidence of WNV among blood donors. 

The role of T-cell immune response against both WNV and USUV is still less known. While CD8 T cells seems to exert a primary role in the clearance of WNV, thus limiting disease severity [19], CD4 T cells contribute to control of WNV by enhancing B-cell response and CD8 T-cell response at late stages of infection [20]. 

In this setting, the aim of our study was to define if WNV or USUV infection was present in a group of 54 blood donors tested positive for WNV NAT in the period 2016–2018 and to evaluate the humoral and cellular response and the duration of humoral immunity for both viruses.

## 2. Materials and Methods

### 2.1. Blood Donors

During the period 2016–2018, out of 73,964 donations tested in Lombardy region, 54 blood donors (10 females and 44 males; median age 47 years, range 20–69) that were WNV-positive by nucleic acid test (NAT) Roche Cobas 6800 (Roche, Rotkreuz, Switzerland) were sent to our Reference Regional laboratory for molecular WNV confirmation. A total number of 275 blood samples were collected during the follow-up (median five samples/blood donor; range 1–11). Blood donors were followed usually until the total disappearance of WNV or USUV IgM; in some cases, follow-up was extended at one or two years to confirm the persistence of immune response. Donors were mainly asymptomatic or paucisymptomatic (low fever and fatigue for few days). The criteria to identify WNV true positive blood donors were as follows: (1) a positive WNV specific reverse transcriptase polymerase chain reaction (RT-PCR) and/or a positive WNV sequence in the pan-Flavivirus nested RT-PCRs independently from serological assay; (2) negative molecular tests but presence of specific WNV IgM, IgG, and neutralizing antibodies (NT Abs) without USUV Abs detection or without USUV NT Abs presence; (3) a four-fold increase in WNV NT Abs titer during the follow-up period compared to USUV titer in cross-reactive serum samples. The same criteria were applied to identify USUV true positive blood donors; however, for molecular assays, the virus was identified only by the pan-Flavivirus nested RT-PCRs followed by sequencing.

### 2.2. Molecular Assays

All the blood samples positive by WNV NAT were tested with our in-house real-time reverse transcriptase polymerase chain reaction (RT-PCR) targeting a conserved region of WNV lineages 1 and 2 [21] and an in-house pan-Flavivirus nested RT-PCRs [15,22], followed by Sanger sequencing of amplicons in case of positive pan-Flavivirus nested RT-PCR results. 

### 2.3. Serological Assays

Serum samples collected during follow-up were tested for the presence of specific IgM and IgG antibodies using enzyme-linked immunosorbent assay (WNV IgM Capture DxSelect^TM^ and WNV IgG DxSelect^TM^ by Focus Diagnostics; Cypress, CA, USA). The same serum samples were tested in parallel for the detection of USUV IgG and IgM antibodies with an indirect immunofluorescence assay (IFA) designed in-house with a serial twofold dilution series starting from 1:10. For detection of IgM antibodies, serum samples were previously absorbed 1:10 with EUROSORB (EUROIMMUN AG, Lubeck, Germany). Briefly, 10 µL of each dilution was added to each spot of a multiwell slide for 30 min at 37 °C in CO_2_. Thereafter, slides were washed, and 10 µL of anti-human IgG or IgM fluorescein antibodies (EUROIMMUN AG) were added for 30 min at 37 °C in CO_2_. After mounting in glycerol, slides were read under fluorescent microscope at 10×. Furthermore, the presence of WNV and USUV neutralizing antibodies (NT Abs) was confirmed by a neutralization assay (NTA) using African green monkey kidney Vero cells (VERO C1008 (Vero 76, clone E6, Vero E6) (ATCC^®^ CRL-1586™)). Briefly, NTA was run starting from 1:10 with a serial fourfold dilution series in microtiter plates with the addition of 50 µL of tissue culture infecting dose 50 (TCID50) of virus, previously titrated, for each dilution. After 1-h incubation at 37 °C, 5% CO_2_, 50 µL of Vero cells were added to each well. Cytopathic effect (CPE) was read after five days of incubation. Serum titer was defined as the highest dilution showing a 50% CPE reduction compared to the virus control. Cross-reactive serum samples in which the NT titer was equal for both WNV and USUV in the 50% CPE NTA were tested also with a 90% CPE reduction NTA in order to increase the specificity. Positive and negative control serum samples were included in each test. Laboratory strains used in this study were the WNV 3B2 strain and the USUV strain Vienna 2001-blackbird (939/01). Antibodies and humoral immunity were detected on consecutive bloods samples over a period of one or two years post infection.

### 2.4. Enzyme-Linked Immunospot Assays

Peripheral blood mononuclear cells (PBMC) from heparinized whole-blood samples, collected at the time of WNV NAT positive results or consecutively, were isolated and used for enzyme-linked immunospot assay (ELISpot assay). In detail, 2 × 10^5^ cells were added in duplicate in Multiscreen-IP membrane-bottomed 96-well plates (Merck Millipore, Darmstadt, Germany) and stimulated with peptide pools (15–18 amino acid length/11 overlap) representative of the E protein of WNV (peptide arrays were obtained by NIH biodefence and Emerging Infections Research resource Repository, NIAID, NIH, Bethesda, MD), at the final concentration of 1 µg/mL. IFN-γ secreting T cells were detected by ex vivo ELISpot assay (Diaclone, Besancon, France), and results were given as net spots/million PBMC as previously described [23]. For USUV-specific immune response, an in-house antigen was produced. In detail, Vero cells were infected with USUV and collected when the CPE was generalized. Medium and cells were harvested, and cells were pelleted at 3000 rpm/10 min, resuspended in 2 mL of the same supernatant and sonicated. The virus was inactivated using an ultraviolet light lamp (UV lamp) for two hours [24]. USUV antigen was titrated in ELISpot assay at different antigen dilution to find the optimal concentration. A dilution of 1:100 of USUV antigen showed the best stimulation of PBMC and was used in all the experiments. 

### 2.5. Ethics Statement

Consent from the local Ethics Committee was not required because, according to a Regional Surveillance and Preparedness Plan (DGR 12591, 27 December 2012), diagnostic detection of WNV infections in the Lombardy Region was centralized at the Regional Reference Laboratory (Molecular Virology Unit, Fondazione IRCCS Policlinico San Matteo, Pavia). Informed consent was not necessary because patients with suspected WNV infections were included in a regional diagnostic protocol. Prospective samples (serum and whole blood) were collected by clinicians and handled by Molecular Virology Unit personnel; data were analyzed anonymously according to a Regional Surveillance and Preparedness Plan (DGR 12591, 27 December 2012).

### 2.6. Statistical Analysis

Descriptive data were reported as absolute and relative frequencies, median, and interquartile range (IQR) based on the type of the variable distribution. For qualitative variables, Fisher’s test was used, while Mann-Whitney test was used for quantitative variables in order to perform comparison between groups. All tests were two-tailed. A *p-*value < 0.05 was considered statistically significant. Analyses were performed using the GraphPad Prism 5 (GraphPad Software, San Diego, CA, USA).

## 3. Results

### 3.1. True Positive WNV and USUV Infections

Among the 73964 blood donations tested, 54 (0.07%) Roche WNV NAT positive blood donors were distributed as follows in the period of follow-up: 14 positive results in 2016, 15 in 2017, and 25 in 2018. Only 12/54 (22.2%) were confirmed as WNV by molecular tests: eight by both WN RT-PCR and pan-Flavivirus nested RT-PCRs (for RT-PCR median 353.7 cp/mL, range 80–1890 cp/mL) and four only by pan-Flavivirus nested RT-PCRs; 6/54 (11.1%) were identified as USUV in the pan-Flavivirus nested RT-PCRs followed by sequencing. Thirty-two donors out of 54 (59.3%) were classified as WNV or USUV according to serological and immunological patterns; in detail, 13/32 cases (40.6%) were classified as WNV and 19/32 (59.4%) as USUV. 

Overall, 25 cases (46.3%) of WNV infection and 25 cases (46.3%) of USUV infection were confirmed. For 4/54 donors (7.4%; two from 2017 and two from 2018), we were not able to perform a differential diagnosis (Table 1) due to the presence of cross-reacting antibodies with the same titer and a short follow-up. 

### 3.2. Serological Patterns of WNV and USUV Infections in Blood Donors

**Figure 1:**
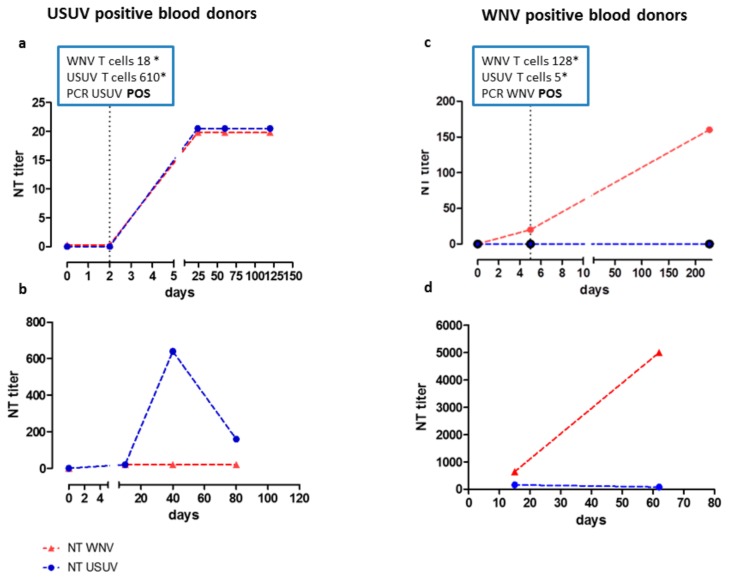
USUV and WNV NT antibodies in serum of two of the 13 blood donors with cross-reacting antibodies. Kinetics of WNV and USUV neutralization titer in two donors with true positive USUV infection followed for several months (**a**,**b**) and in two donors with true positive WNV infection (**c**,**d**) followed for several months are reported. If available, in the box, WNV and USUV ELISpot assay and PCR analysis results are reported. * net spots/million peripheral blood mononuclear cells (PBMC).

The results of serological analysis performed in 50 confirmed blood donors are shown in Table 2. Among the 25 WNV confirmed cases, 15 (60.0%) donors developed only WNV-specific IgG, IgM, and neutralizing (NT) antibodies during the follow-up, whereas, in six donors, cross-reacting antibodies for both viruses were detected and, in four, a previous USUV immunity was present. In the 25 USUV confirmed cases, seven (28.0%) donors developed only IgG, IgM, and NT antibodies for USUV; in eight, cross-reacting antibodies for both viruses were detected, and 10 had a previous WNV immunity. For the 14 donors with cross-reacting antibodies, the use of 90% CPE reduction NT titre was crucial for the diagnosis. Among these 14 donors, some were confirmed also by a positive molecular test and or by a positive ELISpot test. An example of the antibody dynamic in this group of blood donors is given for four blood donors in Figure 1. In panel “a”, we report an example in which WNV and USUV NT Abs maintained the same titer for all the follow-up; in this blood donor, RT-PCR and ELISpot assay were crucial for the USUV diagnosis. In panel “b”, we report a case of USUV infection in which the NTA titer increased only after 40 days. The same trend is reported in Figure 1c,d for WNV cases in which NTA discriminated between the two viruses only after several months, but RT-PCR and ELISpot were immediately informative (Figure 1c). Looking to all 54 positive donors, IgM for WNV, USUV, or both viruses was present at the donation in 19 blood donors and in 31 within the first three weeks (15–20 days).

The NT antibody response to WNV and USUV varied among blood donors, ranging from 1:10 to 1:5000. WNV and USUV IgG and NT antibodies were still present two years after infection on the last tested sample, while IgM antibodies were present up to eight months after onset (data not shown). 

### 3.3. T-Cell Responses against WNV and USUV

WNV-specific T-cell response was measured in six healthy volunteers (WNV−/USUV−), 21 WNV true positive blood donors, and 18 USUV true positive blood donors.

**Figure 2:**
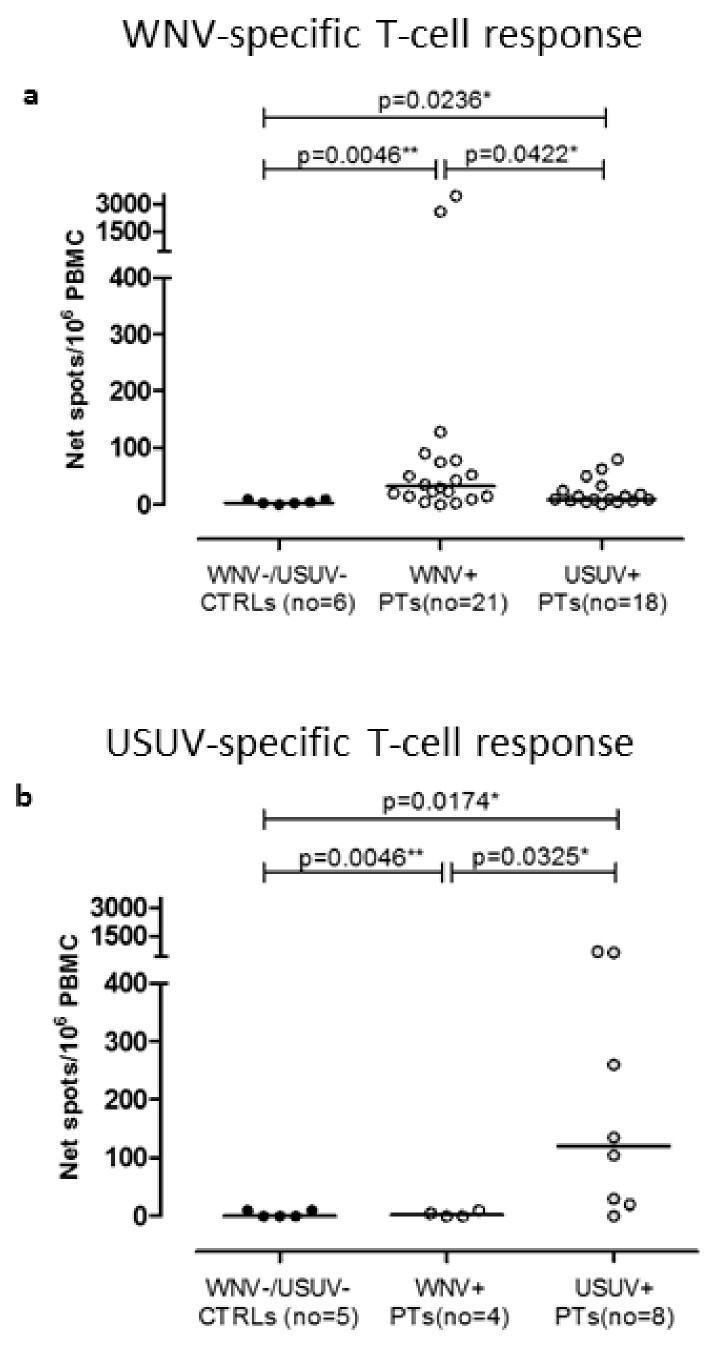
WNV-specific T-cell response was measured in six WNV−/USUV− healthy volunteers (WNV−/USUV− controls (CTRLs)), 21 WNV true blood donors (WNV+ BDs), and 18 USUV true blood donors (USUV+ BDs) (**a**). Similarly, USUV-specific T-cell response was evaluated in five WNV−/USUV− healthy volunteers (WNV−/USUV− CTRLs), four WNV true blood donors (WNV+ BDs), and eight USUV true blood donors (USUV+ BDs) (**b**). Medians of WNV-specific T-cell response are given in the graph for all the three groups of subjects and *p*-values were measured using Mann-Whitney test (* *p* < 0.05; ** *p* < 0.01).

The median WNV-specific T-cell response in WNV−/USUV− healthy volunteers was 4.0 (IQR 2.25–10) net spots/million PBMC, while, in WNV+ and USUV+ blood donors, the median WNV-specific T-cell response was 32.5 (IQR 15–77.25) and 10 (7.5–29) net spots/million PBMC, respectively (Figure 2a). Based on ROC curve analysis, a cut-off of 12.5 net spots/million PBMC of positive WNV-specific T-cell response was calculated (AUC = 0.8917; sensitivity 80%; specificity 100%).

The USUV-specific T-cell response was measured in five healthy volunteers (WNV−/USUV−), four WNV+ blood donors, and eight USUV+ blood donors, due to the low availability of cells. The median USUV-specific T-cell response in WNV−/USUV− healthy volunteers was 0.0 (IQR 0.0–10) net spots/million PBMC, while, in WNV+ and USUV+ blood donors, median USUV-specific T-cell response was 2.5 (IQR 0.0–8.75) and 120 (22.5–522.5) net spots/million PBMC, respectively (Figure 2b). Based on ROC curve analysis, a cut-off of 15 net spots/million PBMC of positive WNV-specific T-cell response was calculated (AUC = 0.9125; sensitivity 87.5%; specificity 100%).

In nine donors (five USUV and four WNV), both WNV and USUV ELISpot assays were performed (Table 3). Interestingly, USUV-specific T-cell response was significantly higher in USUV confirmed cases than in WNV confirmed cases (median 135.0, IQR 67.5–637.5 and median 2.5, IQR 0.0–8.75 net spots/million PBMC, respectively; *p* = 0.0159). However, no difference was observed in terms of WNV-specific T-cell response between USUV confirmed cases (median 18, IQR 11.5–71.5 net spots/million PBMC) and WNV confirmed cases (median 42.5, IQR 16.3–108.5; *p* = 0.7302), suggesting a cross-reaction in terms of T-cell response against E antigen. In 16 blood samples collected for ELISpot determination at the moment of NAT positive detection, a positive antigen-specific T-cell response was detected before the antibody appearance (data not shown).

## 4. Discussion

Since WNV infection became a major problem in endemic areas, entomological surveillance and screening of blood donors were implemented in different regions in northern Italy [6]. Moreover, the emerging problem of USUV co-circulation poses new issues in terms of seroprevalence and cross-reactivity between WNV and USUV. Studies revealed an increase of USUV seroprevalence in northern Italy [25,26,27], especially among forest rangers [28]. In the present study, an increasing number of USUV and WNV cases was reported, in line with other European studies [18,29,30,31,32]. 

Blood donor screening is performed using the WNV NAT test; although this assay is highly sensitive compared to our in-house RT-PCR, it is not able to differentiate between WNV and USUV infection. In the present study, only 33.3% of the WNV NAT positive donors were positive for WNV or USUV with our in-house molecular test, probably due the low number of viral copies at the moment of blood donation. 

Considering the remaining 66.7% of blood donors with undetermined molecular results, serological and/or immunological assays were crucial for establishing which virus was present at the time of NAT positivity. Both humoral and cellular immunity exert an important role in the protection against flavivirus infection. In detail, humoral immunity is responsible for the control of the primary infection and is likely involved in protection against reinfection. 

Infections from closely related flaviviruses, like WNV and USUV, may pose several problems in terms of cross-reactive antibodies that may invalidate the differential diagnosis, as well as several problems for the increased risk of severe disease through a mechanism of antibody-dependent enhancement of infection. 

The main target of flavivirus antibody response is envelope proteins that share similar amino-acid sequences that are responsible for cross-reactivity and cross-immunity [14]. Thus, the detection of neutralizing antibody titer at least fourfold higher for a given flavivirus over the other(s) is considered proof of specificity [33]. Hence, when NT titer is below the threshold, the result has to be considered inconclusive. 

In the absence of positive molecular assays, only using a combination of IgG, IgM, and NT Abs titer calculated on 90% CPE reduction for cross-reacting serum samples, and for some cases a long follow-up (in some blood donors longer than six months as reported in Figure 1b,d), we were able to classify 13 true positive WNV and 18 true positive USUV cases among the 36 undetermined blood donors. In some cases, we found that humoral response to WNV or USUV was still present after two years in contrast with some studies reporting that WNV or USUV-specific antibodies disappear after a few months [26]. Looking at our serological results, if both WNV and USUV cross-reacting neutralizing antibodies are present, the diagnosis of WNV or USUV cases depends on NT titer monitored for at least six months, as reported in Figure 1b,d. In these cases, because the infection occurred at the end of vector season activity, we were confident that we could exclude a new infection.

Finally, in the case of the four true positive WNV cases with a previous USUV infection and 10 true positive USUV cases with a previous WNV infection, no IgM was detected for the previous infection. An explanation for this finding could be that in the context of sequential flavivirus infections, IgM levels are strongly reduced to non-detectable levels [34]. The immune response to primary flavivirus infections is usually characterized by a rapid rise of specific IgM antibodies, which appear to play an important protective role in the early phase of infection [35]. In sequential infections, however, the extent of IgM antibody formation may be reduced and/or delayed [36]. Furthermore, in patients with Zika virus infection and previous DENV immunity, a similar pattern was described [37]. This phenomenon, commonly known as “antibody-dependent enhancement” (ADE), is associated with an increased risk of developing a severe disease and death. However, in our cases, a secondary WNV or USUV infection in the presence of previous WNV or USUV immunity did not worsen the disease.

The high cross-reactivity problem, the low availability of USUV commercial ELISA kits, and the lack of a NAT test that can detect separately the two viruses revealed an important gap in the diagnosis of USUV, which is deeply underestimated.

Focusing on T-cell-mediated response, our new ELISpot assay showed elevated specificity for both WNV and USUV. Interestingly, we observed that the USUV-specific T-cell response, which was high in USUV-positive blood donors, was almost undetectable in WNV-positive blood donors. Although the WNV-specific T-cell response was not highly specific, it was diagnostic for true positive WNV cases. One possible objection is that the use of the antigen E only for the evaluation of WNV-specific T-cell response was not highly specific and that whole WNV lysate should be used. Unfortunately, we could perform USUV- and WNV-specific ELISpot assays only in nine donors. In all these cases, consistent agreement between serological, immunological, and molecular results was observed and, in some cases, ELISpot assays allowed, together with molecular assay, the prompt identification of the virus involved in the infection. As reported in Figure 1a, only NT Abs, although the follow-up was longer than four months, were not informative; in this case, molecular and immunological assays were crucial for the diagnosis of USUV infection. On the other hand, as reported in Figure 1c, NT Abs reached a significant titer for the diagnosis of a WNV true positive case after six months, whereas both RT-PCR and ELISpot assays were suggestive of WNV infection after only five days of follow-up. Interestingly, the positive results detected in the 16 samples collected before antibody appearance gave great value to this new ELISpot tests in the differential diagnosis between these two highly cross-reacting flaviviruses. Moreover, in one case, ELISpot assay was crucial for the diagnosis of USUV infection, in the absence of other positive results. 

In addition to the great potential role of immunological assays for the rapid diagnosis of emerging virus infections, further studies should be performed in a larger sample of patients in order to confirm the data obtained. Moreover, since we reported an unexpected high prevalence of USUV infection among blood donors, blood transfusion services and public health authorities should be aware of a possible increase in human USUV infections.

## Figures and Tables

**Table 1:** Molecular, serological, and immunological results in 54 blood donors distributed for each year of observation. WNV—West Nile virus; USUV—Usutu virus.

Year	WNV Confirmed Cases	WNV RT-PCR/PAN pos	WNV SER pos	USUV Confirmed Cases	USUV PAN pos	USUV SER pos	USUV ELISpot *n* = 9	Unknown (*n*)	Total no. of Blood Donors/Year
2016	9	3	6	5	0	5	0	0	14 (25.9%)
2017	2	2	0	11	6	5	1	2	15 (27.8%)
2018	14	7	7	9	0	9	0	2	25 (46.3%)
total	25 (46.2%)	12 (11.8%)	13 (24.0%)	25 (46.2%)	6 (11.1%)	19 (35.1%)	1 (11.1%)	4 (7.4%)	54

Abbreviations: WNV RT-PCR/PAN pos: blood donors positive for WNV RT PCR or/and pan-Flavivirus nested RT-PCR; WNV SER pos: blood donors positive for WNV serology; USUV PAN pos: blood donors positive for pan-Flavivirus nested RT-PCR; USUV SER pos: blood donors positive for USUV serology.

**Table 2:** Antibody patterns in 50 confirmed blood donors: comparison between true positive WNV- and true positive USUV-positive blood donors.

Serological Patterns	True Positive WNV WNV IgM+, IgG+, NT+	True Positive USUV USUV IgM+, IgG+, NT+	*p*-Value (Fisher’s Exact Test)
USUV IgM-, IgG-, NT-	15	0	<0.0001 *
USUV IgM+, IgG+, NT+	6	0	0.0223 *
USUV IgM-, IgG+, NT+	4	0	0.1099
WNV IgM-, IgG-, NT-	0	7	0.0096 *
WNV IgM+, IgG+, NT+	0	8	0.0040 *
WNV IgM-, IgG+, NT+	0	10	0.0006 *
Total	25	25	

Abbreviations: WNV: West Nile Virus; USUV: Usutu Virus; NT: neutralization; *p*-value was calculated using Fisher’s exact test; * *p*-value significant.

**Table 3:** ELISpot comparative results in five USUV true positive and four WNV true positive blood donors.

Blood Donor	WNV ELISpot Net Spots/Million PBMC	USUV ELISpot Net Spots/Million PBMC	Molecular Test	WNV NT Abs	USUV NT Abs	True Positive for WNV/USUV
1	80	135	USUV	<10	40	USUV
2	8	655	N	10	10	USUV
3	63	105	N	10	40	USUV
4	18	610	USUV	20	20	USUV
5	15	30	N	<10	80	USUV
6	128	5	WNV	160	<10	WNV
7	20	10	WNV	80	20	WNV
8	35	10	N	80	<10	WNV
9	10	0	WNV	40	<10	WNV

Abbreviations: WNV: West Nile virus; USUV: Usutu virus; NT Abs: neutralizing antibodies; PBMC: peripheral blood mononuclear cells.
